# Radiation Exposure of Peripheral Mononuclear Blood Cells Alters the Composition and Function of Secreted Extracellular Vesicles

**DOI:** 10.3390/ijms21072336

**Published:** 2020-03-27

**Authors:** Simone Moertl, Dominik Buschmann, Omid Azimzadeh, Michael Schneider, Rosemarie Kell, Klaudia Winkler, Soile Tapio, Sabine Hornhardt, Juliane Merl-Pham, Michael W. Pfaffl, Michael J. Atkinson

**Affiliations:** 1 Helmholtz Zentrum München, German Research Center for Environmental Health, Institute of Radiation Biology, 85764 Neuherberg, Germany; omid.azimzadeh@helmholtz-muenchen.de (O.A.); michael.schneider@helmholtz-muenchen.de (M.S.); rosemarie.kell@helmholtz-muenchen.de (R.K.); klaudia.winkler@helmholtz-muenchen.de (K.W.); soile.tapio@helmholtz-muenchen.de (S.T.); atkinson@helmholtz-muenchen.de (M.J.A.); 2 Federal Office for Radiation Protection, 85764 Oberschleißheim, Germany; shornhardt@bfs.de (S.H.); michael.pfaffl@wzw.tum.de (M.W.P.); 3 Division of Animal Physiology and Immunology, TUM School of Life Sciences Weihenstephan, Technical University of Munich, 85354 Freising, Germany; dbuschm1@jhmi.edu; 4 Helmholtz Zentrum München, German Research Center for Environmental Health, Research Unit Protein Science, 80939 München, Germany; juliane.merl@helmholtz-muenchen.de; 5 Chair of Radiation Biology, Technical University of Munich, 80333 Munich, Germany

**Keywords:** extracellular vesicles, microRNA, proteome, apoptosis, ionizing radiation, endothelial cells

## Abstract

Normal tissue toxicity is a dose-limiting factor in radiation therapy. Therefore, a detailed understanding of the normal tissue response to radiation is necessary to predict the risk of normal tissue toxicity and to development strategies for tissue protection. One component of normal tissue that is continuously exposed during therapeutic irradiation is the circulating population of peripheral blood mononuclear cells (PBMC). PBMCs are highly sensitive to ionizing radiation (IR); however, little is known about how IR affects the PBMC response on a systemic level. It was the aim of this study to investigate whether IR was capable to induce changes in the composition and function of extracellular vesicles (EVs) secreted from PBMCs after radiation exposure to different doses. Therefore, whole blood samples from healthy donors were exposed to X-ray radiation in the clinically relevant doses of 0, 0.1, 2 or 6 Gy and PBMC-secreted EVs were isolated 72 h later. Proteome and miRNome analysis of EVs as well as functional studies were performed. Secreted EVs showed a dose-dependent increase in the number of significantly deregulated proteins and microRNAs. For both, proteome and microRNA data, principal component analysis showed a dose-dependent separation of control and exposed groups. Integrated pathway analysis of the radiation-regulated EV proteins and microRNAs consistently predicted an association of deregulated molecules with apoptosis, cell death and survival. Functional studies identified endothelial cells as an efficient EV recipient system, in which irradiation of recipient cells further increased the uptake. Furthermore an apoptosis suppressive effect of EVs from irradiated PBMCs in endothelial recipient cells was detected. In summary, this study demonstrates that IR modifies the communication between PBMCs and endothelial cells. EVs from irradiated PBMC donors were identified as transmitters of protective signals to irradiated endothelial cells. Thus, these data may lead to the discovery of biomarker candidates for radiation dosimetry and even more importantly, they suggest EVs as a novel systemic communication pathway between irradiated normal, non-cancer tissues.

## 1. Introduction

Radiation therapy is a major cancer therapy option, with 50–60% of patients treated using this modality [1]. Toxicity to surrounding normal tissues limits the radiation dose that can be applied to the tumor, often leading to suboptimal tumor control or to serious impairment of the quality of life of survivors [2]. For this reason, a more detailed understanding of the normal tissue response to radiation is necessary to predict the risk of normal tissue toxicity and to develop strategies for tissue protection.

One component of normal tissue that is continuously exposed during therapeutic irradiation is the circulating population of peripheral blood mononuclear cells (PBMC). In radiobiology they are often used as a surrogate tissue for damage-based radiation biodosimetry (IAEA 405, Vienna 2001) or they are investigated as predictors of normal tissue radiation sensitivity [3]. PBMCs are particularly sensitive to ionizing radiation (IR) [4,5,6]. Functionally, irradiation of PBMCs leads to imbalances in the immune system and is related to wound healing and inflammation [7,8]. The interaction between PBMCs and endothelial cells was previously identified as a key process in IR-induced inflammation [9,10].

IR exposure results in damage to all cellular macromolecules, however, radiation affects also intercellular communication via gap junctions and release of soluble factors into the extracellular space where they act in an autocrine and paracrine manner (Bystander effect) [11,12]. For example, IR exposure of PBMCs triggers TNFalpha, IL-1 or IFN-gamma release [13,14,15].

Extracellular vesicles (EVs) are increasingly recognized as important mediators in intercellular communication. EVs are a family of different sized membrane-enclosed vesicles secreted from the cell surface, and include exosomes, microvesicles and apoptotic bodies. Their cargo includes proteins, nucleic acids and lipids [16,17]. Once released, EVs can participate in communication with adjacent or distant cells, either by acting as ligands for cell surface structures or by internalization leading to the release of their cargo into recipient cells [18]. EVs are described as an integral part of cellular stress response, including radiation exposure [19,20,21]. Changes in both the cargo and function of EVs released from stressed donor cells have been described [22,23]. First studies have described radiation-induced changes in both the protein and RNA cargo of EVs from a range of cancer cells including glioblastoma [24], lung carcinoma [25] and head and neck cancer [26,27]. Increased survival of cells co-cultivated with EVs from irradiated donors has also been described in these cells. In addition to a survival benefit the radiation-induced EVs conferred increased motility and therapy resistant phenotypes in recipient cancer cells [24,26]. Mrowczynski et al. and Mutschelknaus et al. showed that radiation-derived EVs increased proliferation and enabled recipient cancer cells to survive radiation in vitro as well as increasing tumor burden and decreasing survival in mouse [21,28].

Yet, little is known about how radiation impacts the EV-response of immune cells to IR, although the immune cells as well as potential recipient cells of released factors are critical targets of normal tissue effects. Therefore, we determined whether IR induces changes in the proteins and microRNA (miRNA) cargo of EVs released from PBMCs after radiation exposure to clinically relevant doses between 100 mGy and 6 Gy. Since endothelial injury is increasingly recognized as a consequence of radiotherapy, we asked whether potential cargo changes have functional consequences in endothelial EV recipient cells.

## 2. Results

### 2.1. Characterization of PBMCs and Released Vesicles after Irradiation

**Figure 1:**
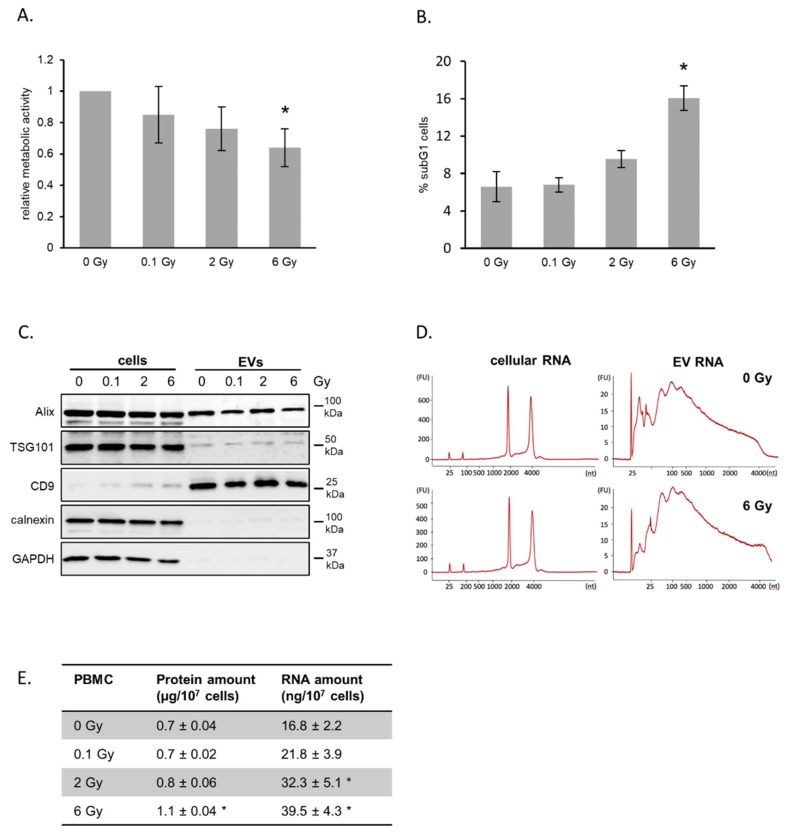
Irradiation of peripheral blood mononuclear cells (PBMCs) and release of extracellular vesicles (EVs). (**A**) Metabolic activity of PBMCs after irradiation. Whole blood was irradiated and PBMCs were isolated and cultivated for 72 h. Mean values from three biological replicates ± SD are shown. (**B**) Quantification of cells in subG1 phase 72 h after irradiation with the indicated doses. Mean values from three biological replicates ± SD are shown. (**C**) Representative Western blot of exosome marker proteins ALIX, TSG101 and CD9 as well as cytosolic markers GAPDH and Calnexin for PBMCs and vesicle lysates 72 h after irradiation. (**D**) Size distribution of total RNA derived from PBMC and PBMC-released EVs determined by Bioanalyzer technology. (**E**) Total protein and RNA yields isolated from EVs released from PBMCs 72 h after irradiation. Proteins were measured by BCA assay and total RNA was quantified by measuring the absorption at 260 nm. Mean values from three biological replicates ± SD is shown. * *p* ≤ 0.05 (calculated by two-sided Student’s t-test).

Whole blood was ex vivo irradiated with 0.1, 2 and 6 Gy, and PBMCs were cultivated in growth medium for 72 h. Cell viability and cell cycle analysis detected a moderate decrease in metabolic activity in response to radiation exposure combined with a moderate dose-dependent increase in subG1 phase cells (Figure 1A,B).

Hence, we concluded that the radiation doses used in this experiment induced moderate acute effects and that the majority of cells were viable at the timepoint of EV isolation. Our EV isolation procedure consisting of differential centrifugation and ultrafiltration enabled the preparation of small EVs, which was confirmed by electron microscopy imaging (EMI), nanoparticle tracking analysis (NTA) and immunoblotting of marker proteins (Figure S1A,B; Figure 1C). EMI showed a cup-shaped structures with a diameter around 120 nm. NTA confirmed a rather homogenous preparation with a mode diameter of 133 nm for EVs from non-irradiated donors and 128 nm for 6 Gy irradiated donor cells (Figure S1B). The detection of the typical marker proteins Alix, TSG101 and CD9 further verified the presence of EVs in our preparations. GAPDH, a protein found in EVs from several cell types [29] was not found in EVs from PBMCs. Calnexin, a component of the endoplasmic reticulum, was not found in vesicle preparations, although it was detectable in cell extracts. This demonstrated the high purity of the EV preparations. The quantification of total RNA and proteins released in vesicles showed for both increasing amounts with increasing exposure of the donor cells, suggesting an increased vesicle release after radiation exposure (Figure 1E). Comparison of total RNA profiles of PBMCs and their vesicles demonstrated the different composition of cellular and vesicular RNA content (Figure 1D). The RNA derived from PBMC-released vesicles was enriched in shorter RNA species, while RNAs from PBMCs dominantly displayed ribosomal RNA.

### 2.2. IR Induces Changes in the EV microRNA Cargo

To identify radiation-induced changes in the microRNA cargo of PBMC-based EVs, we performed small RNA sequencing of RNA preparations derived from pooled PBMC EVs after ex vivo irradiation of whole blood. We applied a pooling strategy to increase the probability for the identification of common radiation-induced changes while reducing individual variabilities.

**Figure 2:**
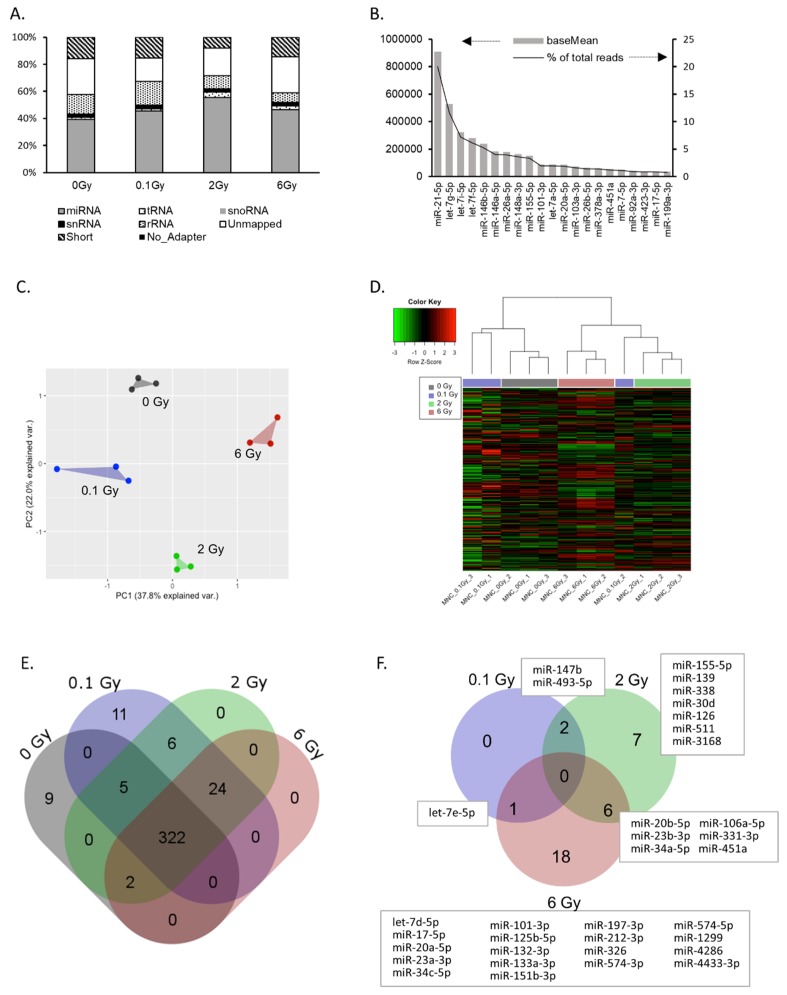
microRNA cargo of extracellular vesicles (EVs) derived from peripheral blood mononuclear cells (PBMCs). (**A**) Percentage of total reads mapped to non-coding small RNAs identified by small RNA sequencing. Short—sequence is shorter than 15 nt; unmapped—sequence did not align to human rRNA, snRNA, snoRNA, tRNA or miRNA. (**B**) Top 20 highly-expressed miRNAs in PBMC EVs (reads per million—RPM, data are expressed as mean mapping percentages). (**C**) Principle component analysis based on the reads of all identified miRNAs in EVs. (**D**) Hierarchical clustering of EV miRNA expression. (**E**) Venn diagram showing total and shared numbers of miRNAs identified at the indicated doses. (**F**) Venn diagram showing the number of total and shared miRNAs deregulated by irradiation. miRNAs are identified with a base mean ≥ 50. A log_2_ fold change ≥ |1| together with an adjusted *p*-value ≤ 0.05 are set as criteria for deregulated miRNAs.

Using mapped sequence read counts, we classified the small RNA content of EVs. As illustrated in Figure 2A, 40–50% of all reads could be assigned to microRNAs. The remaining reads consisted of short or unmapped fragments, fragments without adapter, and to a lesser extent of snRNA, snoRNA, tRNA and rRNAs. Radiation exposure of EV donors did not significantly affect this distribution. In total, 379 miRNAs were identified in EVs from PBMCs (≥ 50 reads) (Table S1). The majority of reads accounted for a small subset of miRNAs, indicating an enrichment of specific miRNAs in EVs. For example, more than 20% of all reads belonged to miR-21-5p (Figure 2B). The complete list of the identified miRNAs including their relative abundancies is shown in Table S1. Principal component analysis (PCA) and unsupervised hierarchical cluster analysis of all identified miRNAs (≥ 50 reads) revealed that the EV miRNA cargo was significantly changed by radiation exposure of the donor cells. PCA showed a clear separation of samples based on the four radiation doses used (Figure 2C). In addition, the hierarchical cluster analysis also revealed dose-dependent miRNA expression changes in EVs. Here, 2 and 6 Gy samples were clearly separated from the 0 Gy samples, while the lower dose of 0.1 Gy had some overlap (Figure 2D). The Venn diagram (Figure 2E) shows the number of identified miRNAs dependent on the dose. Interestingly, several miRNAs were only detected at specific doses. In this way, nine miRNAs were only detected in samples from non-irradiated donors, while 11 miRNAs were exclusively present after low dose irradiation and 24 miRNAs after exposure to either 0.1, 2 or 6 Gy. miRNAs with a fold change of ≥ ±2 (and ≥ 50 reads) compared to 0 Gy were considered to be radiation-regulated and are provided in Figure 2F and Table S2. Comparison of the number of regulated miRNAs dependent on the dose showed an increasing number of regulated miRNAs with increasing dose (Figure 2F). For validation by qPCR, we selected eight miRNAs based on radiation-induced changes and read counts. The significant upregulation of miRNAs miR-34c-5p, miR-34a-5p and miR-20a-5p, as well as the downregulation of miR-574-5p and miR-451 in EVs after irradiation was confirmed (Figure S2). Subsequently, the expression of these miRNAs was analyzed in PBMCs after irradiation. The results suggested that the regulation of a microRNA in the EV was not necessarily correlated to a regulation in the donor cells (Figure S2). Taken together, these results indicate that radiation induces alterations in the microRNA cargo of EVs released by PBMCs and that these changes are dose dependent.

### 2.3. IR Induced Changes in the EV Proteome Cargo

Using label-free quantitative proteomics, we investigated the proteome composition of PBMC-derived EVs from both irradiated and non-irradiated samples. A total of 606 proteins were identified with ≥ two unique peptides (Table S4). These proteins include 89 of the top 100 identified extracellular vesicle proteins summarized in the Vesiclepedia database [29], supporting that there is a largely conserved subset of EV proteins across different cell types.

Of the quantified proteins, 19, 130 and 240 proteins were significantly changed in expression (2 unique peptides; fold change > 1.3 or < 0.7; q < 0.05) after exposure to doses of 0.1, 2 and 6 Gy, respectively (Table S5).

**Figure 3:**
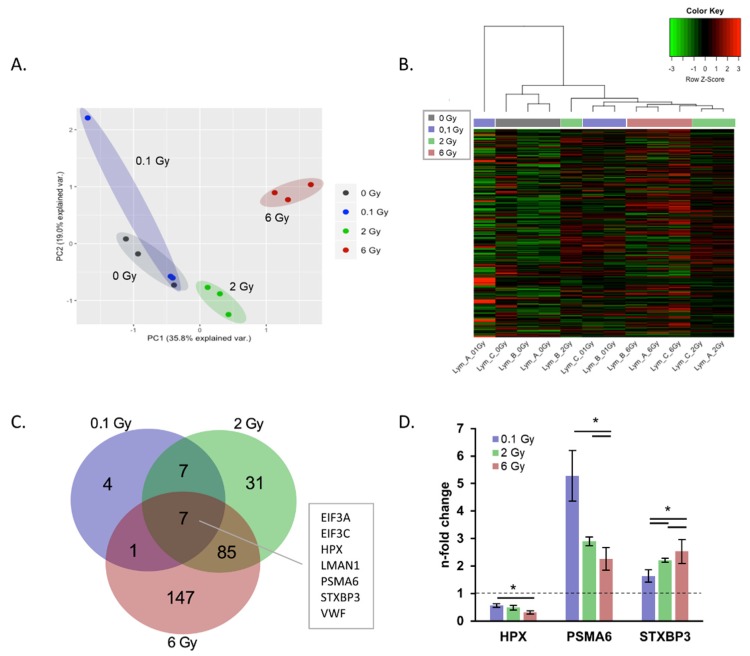
Protein cargo of extracellular vesicles (EVs) derived from peripheral blood mononuclear cells (PBMCs) after irradiation. (**A**) Principle component analysis of radiation effects on the EV proteome based on normalized abundancies of all identified proteins. (**B**) Hierarchical cluster analysis of EV protein expressions in samples from irradiated and non-irradiated donors. (**C**) Venn diagram showing the number of total and shared significantly proteins deregulated after irradiation. (**D**) n-fold changes of dose-dependent regulated EV proteins hemopexin (HPX), proteasome subunit alpha type-6 (PSMA6) and syntaxin-binding protein (STXBP3). * *p* ≤ 0.05 (calculated by two-sided Student’s t-test).

The PCA analysis of all identified proteins showed the separation of EVs from 2 and 6 Gy irradiated donor cells from 0 Gy EVs. The EVs from 0.1 Gy exposed cells were not well separated from 0 Gy (Figure 3A).

The differences in protein expression between the doses are illustrated by a heatmap of all identified proteins including a hierarchical cluster analysis according to the dose (Figure 3B). As seen for the miRNA cargo, the number of significantly deregulated proteins changes dose-dependently following radiation exposure (Figure 3C). The majority of deregulated proteins showed similar direction of fold changes, with the exception of SLC7A5, TMED10 and SNRNP200 which were oppositely regulated at 2 and 6 Gy.

Seven significantly deregulated proteins shared at all three doses (Figure 3C), whereby HPX, PSMA6 and STXBP3 exhibited dose-dependent changes (Figure 3D). Interestingly, the number of regulated proteins was increased with escalating doses and also the adjacent doses of 0.1 and 2 Gy as well as 2 and 6 Gy showed considerable overlap in regulated proteins (Figure 3C).

### 2.4. Functional Annotation of Radiation-Regulated EV Components

**Figure 4:**
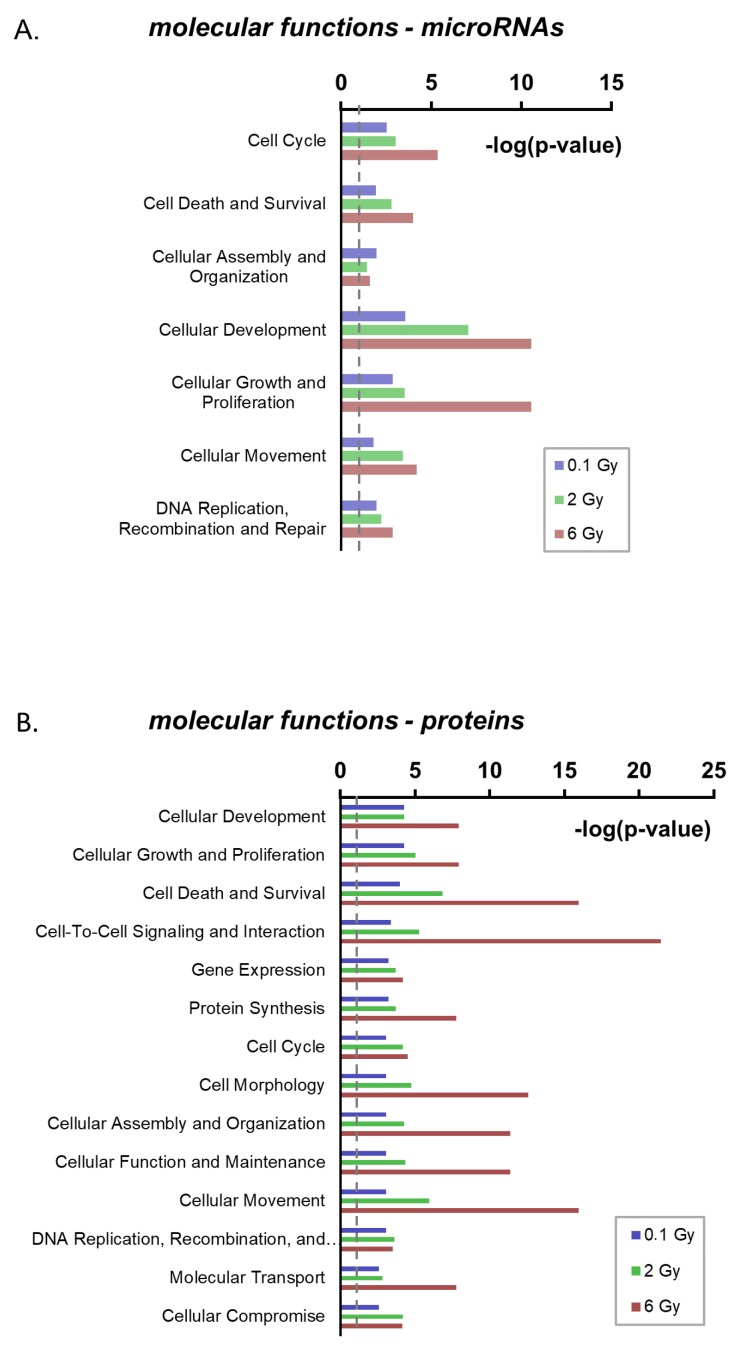
Ingenuity Pathway Analysis of radiation deregulated microRNAs and proteins in extracellular vesicles. (**A**) Enrichment analysis of molecular functions for radiation-deregulated miRNAs. (**B**) Enrichment analysis of molecular functions for radiation-deregulated proteins. Only pathways significantly enriched at all three doses are shown. (dashed line displays *p* = 0.05).

In order to identify potential functional consequences of radiation-induced alterations in the EV cargo, the predicted molecular functions of the deregulated miRNAs and proteins were analyzed by an integrative analysis (Ingenuity Pathway Analysis (IPA), http://www.ingenuity.com) (Table S3). For the regulated miRNAs, the IPA analysis identified seven categories of molecular functions that might be affected at all three doses. These included typical radiation response pathways such as cell cycle, cell death and survival, DNA replication, recombination and repair (Figure 4A). Within these pathways, apoptosis, as a subpathway in cell death and survival, showed the lowest activation z-score (−2.8), suggesting a potential suppressive function of the deregulated EV miRNAs on apoptosis once they have entered recipient cells (Figure S3).

Functional enrichment analysis of radiation-regulated proteins resulted in the identification of 14 processes potentially affected by the changed candidates following exposure to all three doses. These included the same radiation response pathways identified for deregulated miRNAs (Figure 4B, Table S6). Furthermore, analysis of deregulated proteins predicted a significant inactivation of cellular apoptosis following exposure to the dose of 2 and 6 Gy (z-Scores: −1,9 (2 Gy); −3,3 (6 Gy)) (Figure S4A,B). Together these predictions suggest that both, radiation-regulated microRNAs and proteins in EVs, have overlapping or cooperative functions in the radiation response.

### 2.5. Functional Analysis of EVs Released from Irradiated Donors: Preferential Targeting of Endothelial Cells

**Figure 5:**
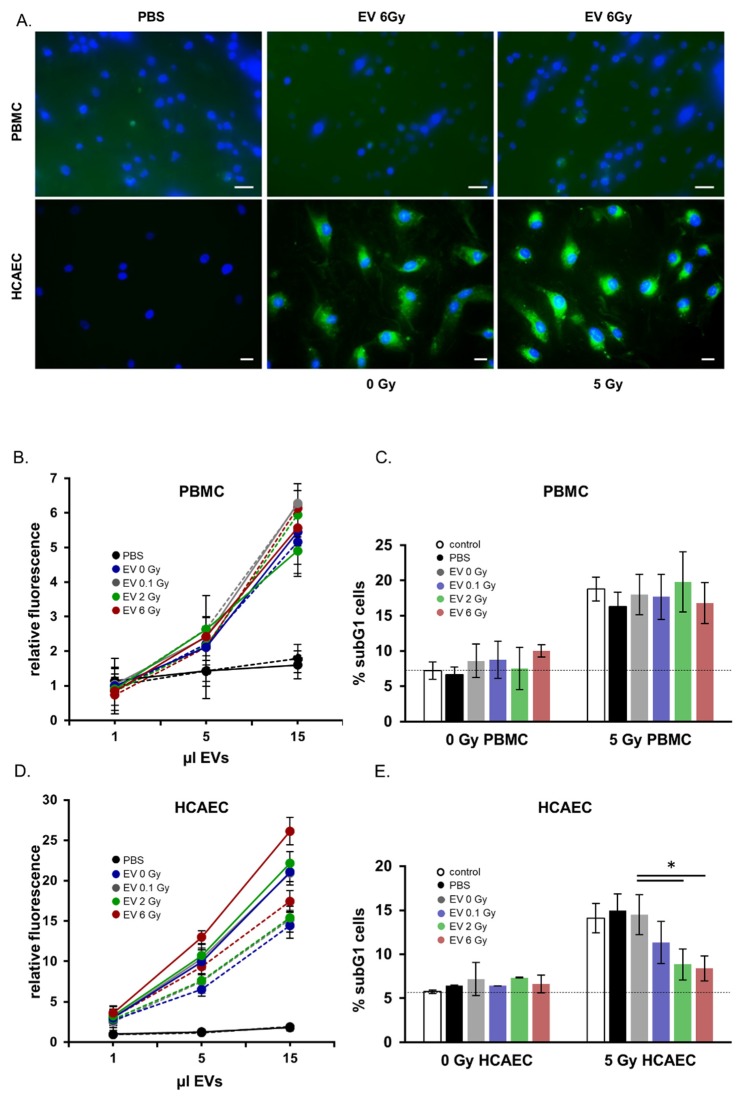
Functional analysis of extracellular vesicles (EVs) derived from peripheral blood mononuclear cells (PBMCs) after irradiation. (**A**) Immunofluorescence microscopy images of EV incorporation in PBMCs and in human endothelial cells. Nuclei are in blue, EVs appear in green (scale bar: 20 µm). (**B**) Quantification of PBMC EV-uptake in PBMCs by flow cytometry. The uptake was monitored after 24 h of co-cultivation. Solid lines show irradiated recipient cells, dashed lines display non-irradiated recipient cells. (**C**) Quantification of apoptosis measured by subG1 cells in PBMCs after co-cultivation with EVs from irradiated and non-irradiated PBMC donors. (**D**) Quantification of PBMC EV-uptake in human coronary artery endothelial cells (HCAEC) by flow cytometry. The uptake was monitored after 24 h of co-cultivation. Solid lines show irradiated recipient cells, dashed lines display non-irradiated recipient cells. Mean values from three biological replicates ± SD is shown. (**E**) Quantification of apoptosis measured by subG1 cells in HCAEC after co-cultivation with EVs from irradiated and non-irradiated PBMC donors. Mean values from three biological replicates ± SD is shown, * *p* < 0.05 (calculated by two-sided Student´s t-test).

In order to analyze the functional properties of EVs released from irradiated or non-irradiated donors, we measured the uptake of EVs in potential recipient cells. Figure 5A depicts representative immunofluorescence images of EV uptake in irradiated and non-irradiated PBMCs and endothelial cells. Uptake and cytoplasmic distribution of the labelled EVs were clearly visible 24 h after EV co-cultivation within the endothelial recipient cells. Uptake in PBMCs was less pronounced. The quantitative analysis of EV uptake in recipient cells by flow cytometric analysis is shown in Figure 5B,D. These results confirmed our microscopy observations (Figure 5A), indicating a more efficient uptake in endothelial cells than in PBMCs as well as an enhanced uptake with increasing amounts of added EVs. The effect of radiation on the uptake of EVs by recipient cells was investigated by comparing the uptake kinetics of EVs in non-irradiated and 5 Gy irradiated recipient cells. Figure 5C shows an increase of the uptake of EVs in irradiated endothelial recipient cells compared to that by non-irradiated cells. This was consistently found for EVs isolated from the irradiated and the non-irradiated donors.

However, there was no significant difference in the kinetics of uptake of EVs between the irradiated or non-irradiated PBMCs (Figure 5B). Also the irradiation status of the EV donors showed no influence on the uptake.

Based on the predicted impact of EVs on the apoptosis in recipient cells we preincubated PBMCs or endothelial cells with EVs isolated from either non-irradiated (EV 0 Gy) or irradiated (EV 0.1 Gy, EV 2 Gy, EV 6 Gy) PBMCs. Twenty-four hours later the recipient cells were irradiated with 5 Gy and cell cycle analysis was performed 48 h later. The co-cultivation of EVs had no effect on the amount of subG1 cells in non-irradiated PBMCs and endothelial cells (Figure 5C,E). However, preincubation with EVs isolated from 2 (EV 2 Gy) and 6 Gy (EV 6 Gy) irradiated donors significantly reduced the amount of cells in subG1 phase after irradiation compared to a control (PBS) preincubation or an incubation with EVs from non-irradiated donors (EV 0 Gy) in endothelial cells (Figure 5E). An EV treatment had no effect on subG1 phase cells neither on irradiated nor on non-irradiated PBMCs (Figure 5C). In accordance with reduced subG1 fractions in irradiated HCAEC cells co-cultivated with EVs from 2 and 6 Gy irradiated PBMCs, we detected decreased caspase-3 activity in these cells (Figure S5).

## 3. Discussion

Extracellular vesicles (EVs) are potential mediators of the cellular stress response [30,31]. Our study identified EVs as part of the communication between irradiated PBMCs and endothelial cells. We have shown that the radiation exposure affects both, the level and composition of EVs secreted from PBMCs, as well as the ability of endothelial cells to take up EVs. Moreover, we have identified an apoptosis-suppressive effect of EVs from irradiated donors (2 and 6 Gy) in irradiated endothelial cells. Thus our results suggest that EV-based communication is responsible for a prosurvival signaling between normal tissues in response to irradiation.

### 3.1. Radiation Induces Changes in the miRNA and Protein Cargo of EVs

To identify radiation effects on EVs secreted from PBMCs, we used ex vivo-irradiated blood as an exposure model, which was demonstrated to accurately reflect the in vivo peripheral blood radiation response in humans [32]. Our study indicated that IR is able to change the release and content of EVs secreted from PBMCs. As we found a dose-dependent increase in protein and RNA amounts in EVs isolated from irradiated cells, and also increased EV numbers isolated from 6 Gy irradiated cells compared to unirradiated cells (Figure S1C), we suggest an irradiation-induced increase in the release of EVs in irradiated cells. In line with these data, enhanced EV secretion was also reported in glioblastoma and head and neck cancer cells after radiation exposure [21,24].

Using small RNA sequencing and whole proteome analysis, we identified differences in the miRNA and protein composition in vesicles from irradiated versus control cells. Interestingly, EV cargo changes were found to be highly dose-dependent. The number of deregulated miRNAs and proteins was increased with ascending doses, reflecting the enhanced effect on the donor cells. Moreover, most changes were dose specific. None of the miRNAs was changed at all three doses and several miRNAs were exclusively detected in non-irradiated or irradiated cells. Out of the 283 significantly-changed proteins, only seven were changed at all doses, whereby three proteins exhibited dose-dependent regulations.

Four of the radiation-deregulated EV miRNAs (miR-155-5p, miR-20a-5p, miR-17-5p, miR-451a) may be particularly attractive for future studies, as they were also detected amongst the top 20 most abundant miRNAs. Although their functions were mostly studied in cancer cells all four miRNAs were shown to be involved in radiation resistance. In hypoxic lung cancer cells an inhibition of miR-155-5p leads to radiosensitization [33]. miR-20a-5p induces radioresistance in hepatocarcinoma [34] and in nasopharyngeal cancer cells [35]. miR-17-5p is a member of the miR-17-92 tumor suppressor cluster, which was shown to be upregulated after irradiation in several cancer and noncancer systems [36]. An overexpression of the cluster confers radioresistance to lymphoma cells [37]. On the contrary, radiosensitizing effects were described for miR-451a in lung cancer [38,39]. Considering the radiation-increased levels of miR-155-3p, miR-20a-5p and miR-17 together with the decreased amount of miR-451a, EVs from irradiated donors have the potential to transport prosurvival signals.

The comparison of intracellular and vesicular miRNA profiles showed that changes in EVs do not reflect intracellular expression changes, which suggests controlled, active packaging mechanisms. However, previously suggested “exo loading motifs” [40] were not enriched in the found miRNA profile (data not shown).

Different to miRNA changes, the proteins hemopexin (HPX), syntaxin-binding protein 3 (STXBP3) and proteasome subunit alpha type-6 (PSMA6) showed dose-dependent changes, which makes them potential candidates for further investigations as biomarkers for radiation response. Hemopexin as well as syntaxin-binding protein are also described as EV components released from cancer and other non-cancer cells [41,42,43,44], but their putative roles in radiation response are so far unclear. The radiation-induced upregulation of PSMA6 is in line with recent findings in EVs from head and neck [26] and glioblastoma cells [28] suggesting that this protein is radiation-regulated in a cell type-independent manner. As a component of the proteasome complex, PSMA6 is involved in proteolytic degradation, which is a critical process in radiation response [45]. Additionally, PSMA6 was described as a DNA damage associated protein, which mediates DNA repair pathways [46].

### 3.2. Physiological Relevance of PBMC-Derived EVs after Ionizing Radiation

EVs can be incorporated into recipient cells, where they have the potential to alter biological functions [17,31]. We identified endothelial cells as a more efficient recipient system for PBMC-EVs than PBMCs themselves, which suggests cell type specificity in EV donor and recipient interactions. Moreover, irradiated endothelial recipient cells were able to incorporate EVs (from irradiated and non-irradiated donors) more efficiently than their non-irradiated counterparts, suggesting that irradiation-induced changes in endothelial cells enhanced EV uptake. In accordance, increased uptake of EVs in irradiated cells was also demonstrated in HNSCC [21] and mesenchymal stem cells [47]. As a potential mechanism, Hazawa et al. suggested an enhanced co-localization of integrin CD29 and tetraspanin CD81 on the plasma membrane [47]. As EVs from irradiated and non-irradiated donors were incorporated with similar efficiency, we concluded that radiation-induced changes to the EVs did not change their uptake properties. Consistent with these results, it was shown in several cancer cell lines that the EV uptake capability was not dependent on the expression of vesicle marker proteins, but on the recipient cells [48,49].

After assessing EV incorporation, we investigated putative EV-induced phenotypic changes in PBMCs and endothelial cells. Co-cultivation of EVs has no effect on apoptosis in non-irradiated PBMCs and endothelial cells. But, our results demonstrated lower levels of apoptosis in irradiated endothelial cells when they were preincubated with EVs from 2 and 6 Gy irradiated donors, suggesting anti-apoptotic functions for these EVs. EVs released after low-dose irradiation (0.1 Gy) showed no effect on apoptosis. These findings suggest a cell type-specific, pro-survival role of EVs during the cellular radiation response, while there was no effect in non-stressed cells. This finding for EVs from irradiated PBMC donors stand in line with results obtained in several tumor cell models, where EVs from irradiated donors also conferred prosurvival signals to recipient cells [21,24,28,47]. However, this pro-survival effect of EVs from irradiated donors was not found in PBMCs, which suggests cell type-specific functions of EVs. Considering the low uptake efficiency of EVs in PBMC recipient cells, we speculate that EV uptake is an important regulator of cell-type specific EV signaling.

Taking into account that EV-shuttled miRNAs and proteins potentially trigger phenotypes in recipient cells [42,50] and that in silico analysis of our RNA and protein data suggested that irradiation-stressed PBMCs secrete EVs with upregulated anti-apoptotic and downregulated apoptosis-promoting cargo, we propose the following model:

Irradiation of PBMCs induces the release of EVs with an increased anti-apoptotic cargo. Simultaneously, irradiation enhances the uptake of EVs into endothelial cells. EV-transferred proteins and miRNAs then contribute to the suppression of apoptosis in the irradiated endothelial cells. Considering the moderate n-fold changes but the large number of apoptosis mediator components within the radiation-changed EV cargo, we argue that the combined activity of multiple factors may be responsible for the observed effect. miR-23a, miR-451a and miR-101-3p are interesting candidates to contribute to suppressed apoptosis in endothelial recipient cells [51,52]. These miRNAs are decreased (miR-23a-3p, mir-451a) or increased (miR-101-3p) in EVs from irradiated donors, and corresponding regulations were previously shown to suppress apoptosis in endothelial cells [51,53,54,55].

From the clinical perspective endothelial cell apoptosis is responsible for acute normal tissue radiation damage, especially in the gastrointestinal tract, hematopoietic system and lungs [56,57]. As a consequence, therapeutic anti-tumor radiation doses are limited by the increased killing of endothelial cells. Our study demonstrated that the EV-mediated communication between irradiated PBMCs and endothelial cells is a part of the cellular radiation response that has the potential to suppress radiation-induced endothelial cell killing, and thus to counteract normal tissue toxicity. Thus, our data contribute to a better understanding of the radiosensitivity in endothelial cells, which will contribute to innovative strategies for improved radiation therapy.

## 4. Materials and Methods

### 4.1. Blood Collection, Irradiation and Cell Culture

All experimental procedures were carried out according to the local ethical standards in agreement with the standards of the declaration of Helsinki of the World Medical Association. Written informed consent and consent to publish was obtained from the participants.

Blood was collected from five healthy female donors aged between 30 and 50 in BD Vacutainer Plus plastic whole blood tubes with spray-coated K_2_EDTA (reference no. 367525, BD Biosciences, Heidelberg, Germany) and immediately processed. The collected whole blood was irradiated with 195 kV/10 mA X-rays using a 3.0 mm aluminum filter at a dose rate of 0.82 Gy per minute (X-Strahl RS225). After irradiation, peripheral blood mononuclear cells (PBMC) were separated by Ficoll-Paque™ PLUS (GE Healthcare, Uppsala, Sweden) centrifugation and cultivated for 72 h at 37 °C in RPMI1640 (Thermo Fisher Scientific, Dreieich, Germany) supplemented with 10% EV-depleted fetal calf serum (FCS) and 5 µg/mL PHA (Sigma-Aldrich, Schnelldorf, Germany). Subsequently, conditioned medium and PBMCs were separated by centrifugation at 300× *g* for 10 min and stored at –80 °C for further steps.

To measure metabolic activity of PBMC isolated after whole blood irradiation, 5000 cells were incubated in 100 µL RPMI1640 (Thermo Fisher Scientific) supplemented with 10% EV-depleted fetal calf serum (FCS) and 5 µg/mL PHA (Sigma-Aldrich) in 96-well plates. Seventy-two hours after radiation exposure, 100 µL of CellTiter-Glo Luminescent Cell Viability Assay reagent (Promega, Walldorf, Germany) was added and luminescence was measured with a plate reader (TECAN, Männedorf, Switzerland).

The human coronary artery endothelial cells (HCAECs; ATCC^®^ PCS-100-020™) were cultivated in MesoEndo Cell Growth Medium Kit (Cell Applications, San Diego, CA, USA) at 5 % CO_2_. Settings for irradiation were identical to the irradiation of whole blood (see above). Irradiation parameters at the X-Strahl RS225 were routinely checked by using a UNIDOS E dosimetry system (PTW, Freiburg, Germany).

### 4.2. EV Isolation

For proteomics, RT-qPCR and all functional assays, EVs were isolated from the culture supernatants by serial high-speed centrifugation procedure, as previously described [21,26]. Briefly, EVs were isolated from 2 × 10^7^ PBMCs seeded in a 75 cm^2^ cell culture flask in fresh medium with EV-depleted FCS. After 72 h of cultivation, the medium was collected, centrifuged at 10,000× *g* for 30 min at 4 °C and passed through a filter with a pore size of 0.22 µm. The filtrate was centrifuged at 100,000× *g* for 75 min at 4 °C in a Type 70.1 Ti fixed-angle titanium rotor (Beckmann Coulter, Krefeld, Germany). The supernatant was discarded and the EV pellet was resuspended in PBS. Another round of ultracentrifugation (100,000× *g*, 75 min, 4 °C) was applied and the final EV pellet was resuspended in 20 µL PBS.

For small RNA sequencing, the conditioned medium was centrifuged at 10,000× *g* for 30 min at 4 °C, concentrated to 1/10 of the original volume by ultrafiltration (Amicon Ultra, 10k MWCO, Merck-Millipore, Darmstadt, Germany) and then passed through a filter with a pore size of 0.22 µm. Subsequently, EVs were precipitated using the miRCURY Exosome Isolation Kit (#300102, Exiqon, Vedbaek, Denmark) according to the manufacturer’s instructions. All EVs were stored at −80 °C. PBMCs were harvested using a cell scraper and stored at −20 °C. For the preparation of EV-depleted FCS, bovine EVs were removed from FCS by centrifugation at 100,000× *g* and 4 °C for 14 h.

### 4.3. Electron Microscopy

PBMC-derived EVs were absorbed onto glow discharged carbon coated grids (G2400C from Plano) for 2 min. The solution was blotted of and negatively stained with 4% ammonium molybdate (Sigma-Aldrich) solution for 30 s. Micrographs were recorded with a Jeol JEM 100CX electron microscope at 100 kV onto Kodak SO163 film. Negatives were digitized with a Hasselblad Flextight × 5 scanner at 3000 dpi, resulting in a pixel size of 0.25 nm/px. For visualization images were binned to 1 nm/px.

### 4.4. Particle Size Determination

EV size distribution was analyzed by using the NanoSight LM10 (Malvern Panalytical, Malvern, UK) microscope. EV preparations (isolated from 2.5 mL conditioned medium) were diluted 1:600 with H_2_O to achieve 15 to 50 particles per frame for tracking. Each sample was analyzed three times for 30 s.

### 4.5. Quantitative Proteomic Analysis

EV proteins were isolated by adding 20 µL of lysis buffer (25 mM Tris pH 7.5, 120 mM NaCl, 1 % Triton X-100, 1 % PSMF, 1 mM NOV, 1 mM Leupeptin) to 40 µL of EV suspension isolated from 2 × 10^7^ cells. The samples were incubated for 4 h on ice with repeated vortexing and the protein concentration was determined by the BCA assay (PierceTM BCA Protein Assay Kit, Thermo Fisher Scientific) following the manufacturer’s instructions.

### 4.6. Filter-Aided Sample Preparation (FASP) Digest

For each sample 10 µg EV protein lysate were digested using a modified filter-aided sample preparation procedure [58]. After reduction and alkylation using dithiothreitol and iodoacetamide, the proteins were centrifuged on a 30 kDa cutoff filter device (Sartorius), washed thrice with UA buffer (8 M urea in 0.1 M Tris/HCl pH 8.5) and twice with 50 mM ammonium bicarbonate. The proteins were digested for 2 h at room temperature using 1 µg Lys-C (Wako Chemicals, Neuss, Germany) and for 16 h at 37 °C using 2 µg trypsin (Promega, Germany). After centrifugation (10 min at 14,000× *g*), the eluted peptides were acidified with 0.5% TFA and stored at −20 °C.

### 4.7. LC-MSMS Measurements

LC-MS/MS analysis was performed on a Q-Exactive HF mass spectrometer (Thermo Scientific Scientific) online coupled to an Ultimate 3000 nano-RSLC (Dionex). Tryptic peptides were automatically loaded on a C18 trap column (300 µm inner diameter (ID) × 5 mm, Acclaim PepMap100 C18, 5 µm, 100 Å, LC Packings) at 30 µL/min flow rate prior to C18 reversed-phase chromatography on the analytical column (nanoEase MZ HSS T3 Column, 100Å, 1.8 µm, 75 µm × 250 mm, Waters), at 250 nl/min flow rate in a 95 min non-linear acetonitrile gradient from 3% to 40 % in 0.1% formic acid. Profile precursor spectra from 300 to 1500 m/z were recorded at 60,000 resolution with an automatic gain control (AGC) target of 3e6 and a maximum injection time of 50 ms. TOP10 fragment spectra of charges 2 to 7 were recorded at 15,000 resolution with an AGC target of 1e5, a maximum injection time of 50 ms, an isolation window of 1.6 m/z, a normalized collision energy of 28 and a dynamic exclusion of 30 s.

### 4.8. Quantitative Data Analysis Using Progenesis QI for Proteomics

Generated raw files were analyzed using Progenesis QI for proteomics (version 3.0, Nonlinear Dynamics, part of Waters) for label-free quantification, as described previously [58,59]. Features of charges 2–7 were used and all MSMS spectra were exported as mgf file. Peptide search was performed using Mascot search engine (http://www.matrixscience.com, version 2.5.1) against the Ensembl human protein database (83,462 sequences, 31,286,148 residues). Search settings were 10 ppm precursor tolerance, 0.02 Da fragment tolerance, one missed cleavage allowed. Carbamidomethyl on cysteine was set as fixed modification, deamidation of glutamine and asparagine allowed as variable modification, as well as oxidation of methionine. Applying the percolator algorithm [60] resulted in a peptide false discovery rate (FDR) of 0.54%. Search results were reimported in the Progenesis QI software. Proteins were quantified by summing up the abundances of all unique peptides per protein. Resulting normalized protein abundances were used for calculation of fold-changes and statistical values were exported from the Progenesis QI software. For final quantifications, proteins were identified with at least 2 unique peptides and with ratios greater than 1.3-fold or less than 0.7-fold (q < 0.05) were defined as being significantly differentially expressed.

### 4.9. Data Availability

The raw MS data were deposited in the RBstore database, Study ID: 1155: (https://www.storedb.org/store_v3/create_dataset.jsp?studyId=1155).

### 4.10. RNA Analysis

Total RNA was isolated from EVs isolated from 2 × 10^7^ PBMCs using the miRCURY RNA Isolation Kit (Cat. #300110, Exiqon, Denmark) according to the manufacturer’s protocol. The concentration of RNA samples was measured by absorption at 260 nm using a Nanodrop (Thermo Fisher Scientific, Dreieich, Germany). RNA samples were stored at −80 °C for up to 3 months. To analyze the size distribution and quality of the RNA eluate, 1 μL of the eluate was subjected to the Bioanalyzer RNA 6000 Pico assay on a 2100 Bioanalyzer (Agilent Technologies, Santa Clara, CA, USA) according to the manufacturer’s instructions.

### 4.11. Small RNA Sequencing

Library preparation and small RNA-sequencing were performed as described previously [52]. Briefly, 10–20 ng EV-RNA was dried using a centrifugal evaporator and resuspended in 10 µL nuclease-free water. Six µL of this concentrated RNA was used as input material for library construction, which was carried out utilizing the NEBNext Multiplex Small RNA Library Prep Set for Illumina (New England BioLabs, Ipswich, MA, USA). After 15 cycles of PCR-based amplification, individual libraries were assessed by capillary electrophoresis (DNA1000 Assay, 2100 Bioanalyzer, Agilent Technologies, Santa Clara, CA, USA) and pooled. The cDNA library pools were size selected using high-resolution gel electrophoresis (4% agarose) and regions corresponding to cDNA fragments with inserts in the miRNA size range were collected. After a final quality control step (High-Sensitivity DNA Assay, 2100 Bioanalyzer, Agilent Technologies), libraries were subjected to 50 cycles of single-end sequencing using a HiSeq2500 (Illumina, San Diego, CA, USA).

### 4.12. Sequencing Data Analysis

FastQC (https://www.bioinformatics.babraham.ac.uk/projects/fastqc/) was used to assess sequencing quality and length distribution of inserts. Next, adaptor sequences were clipped using Btrim [61]. Reads without adaptors as well as reads shorter than 16 nt were eliminated from the data set. Additionally, reads that aligned to rRNA, tRNA, snRNA and snoRNA sequences downloaded from RNAcentral [62] were discarded to reduce the likelihood of false positives during miRNA mapping. Remaining reads were aligned to miRBase version 21 [63] using Bowtie [64] and the “best” algorithm. One mismatch per sequence was allowed for all alignment steps. Read counts were generated by summing up all per-sequence hits in Bowtie output.

Differential miRNA expression was assessed using the Bioconductor package DESeq2 (version 1.8.1) for R [65] and the corresponding strategies for normalization (median ratios of mean expression) and false discovery correction (Benjamini–Hochberg). Thresholds for the detection of significantly regulated miRNAs were set to base mean ≥ 50, log_2_ fold change ≥ |1| and p_adj_ ≤ 0.05. Sequencing data were deposited under STUDY PRJEB35150 at https://www.ebi.ac.uk/ena.

### 4.13. qPCR

For qPCR, the TaqMan advanced microRNA assay system (Thermo Fisher Scientific) was used according to the manufacturer’s protocol. Following primers were used: hsa-miR-20a-5p (#478586_mir), hsa-miR-34a-5p (#478048_mir), hsa-miR-34c-5p (#478052_mir), hsa-miR-451a (#478107_mir) and hsa-miR-574-3p (#478163_mir). N-fold changes were calculated by using the 2^−∆∆cT^ method. For normalization, hsa-miR-26a-5p (#477995_miR) was used. According to NGS data analysis, this microRNA was highly expressed in PBMC EVs and not changed by irradiation.

### 4.14. Apoptosis

For the detection of apoptotic cells, HCAEC or PBMC target cells were incubated in 24-well plates in 500 µL EV-depleted medium containing EV suspensions (3 µL) isolated from irradiated and non-irradiated PBMCs. After 24 h preconditioning, cells were irradiated with 0 or 5 Gy. Forty-eight hours after irradiation, cells were harvested. The cell pellet was resuspended in 300 µL of a solution containing 10 mM NaCl, 4 mM Na-citrate, 10 µg/mL RNase A, 0.3% Nonidet P-40 and 50 µg/mL propidium iodide and gently vortexed. The cell suspensions were incubated for 30 min at room temperature followed by the addition of 300 µL of solution containing 70 mM citric acid, 250 mM sucrose and 50 µg/mL PI. The cell suspensions were mixed and stored at 4 °C before flow cytometry. Cell cycle distributions were analyzed on a FACScan LSR II (BD Biosciences, Heidelberg, Germany) (excitation wavelength—488 nm, emission wavelength—610 nm, LSR II, BD/FACS DIVA Software). Cells with a DNA content less than that of cells in the G1 phase of the cell cycle were assigned to the sub-G1 fraction and are considered to be apoptotic.

Caspase-3 activity was monitored by using the caspase-3 substrate Ac-DEVD-pNA (Calbiochem, Darmstadt, Germany) as recently described [54]. Briefly, HCAEC cells were incubated in 24-well plates in 500 µL EV-depleted medium containing EV suspensions (3 µL) isolated from irradiated and non-irradiated PBMCs. After 24 h preconditioning, cells were irradiated with 0 or 5 Gy. Twenty-four hours after irradiation, cells were harvested and caspase-3 activity was monitored using a plate reader (TECAN, Männedorf, Switzerland).

### 4.15. EV Uptake

PBMC-EV staining was performed with the green fluorescent dye PKH67 (MINI67-1KT, Sigma-Aldrich). To this end, 10 µL of EV solution were resuspended in 250 µL of the diluent C plus 1.5 µL PKH67. After 10 min at room temperature, excessive dye was removed by using Exosome Spin Columns (Thermo Fisher Scientific) according to the manufacturer’s protocol. An equal amount of dye in diluent C plus 50 µL of PBS was processed in parallel to EVs as a negative control.

To measure the uptake of EVs, 75,000 PBMC or HCAEC cells were seeded in 24-well plates in 500 µL medium. Twenty-four hours later, cells were irradiated with 5 Gy or sham treated. After another 24 h, equal amounts of PKH67-stained EVs were added to irradiated and non-irradiated recipient cells. After an additional 24 h, the recipient cells were washed three times with PBS, trypsinized and resuspended in 500 µL of PBS. Uptake was quantified on a FACSCAN LSRII (Becton-Dickinson, excitation—490 nm, emission—502 nm). For fluorescence microscopy cells were washed three times with PBS, fixed with 4% paraformaldehyde, washed again with PBS and covered with Vectashield including Hoechst 33342 for nuclear staining. Immobilization of PBMCs on microscopy slides was achieved by a standard cytospin procedure. Pictures were taken with a BZ-9000 fluorescence microscope (Keyence, Osaka, Japan).

### 4.16. Immunoblotting

Cells and EVs were separately disrupted in lysis buffer (25 mM Tris pH 7.5, 120 mM NaCl, 1% Triton X-100, 1% PSMF, 1 mM NOV, 1 mM Leupeptin) for 4 h on ice.

For immunoblotting, 10 µg cellular protein (determined by the BCA assay (PierceTM BCA Protein Assay Kit, Thermo Fisher Scientific)) or 10 µL EV lysate (corresponding to EVs isolated from 3.5 × 10^6^ cells) were used to run a standard denaturing Western blot protocol. Membranes were blocked with Roti-Block (Roth, Darmstadt, Germany) and washed three times with TBS-T after each step. Antibodies directed against ALIX (2171, Cell Signalling, Danvers, MA, USA), TSG101 (GTX70255, GeneTex, Irvine, CA, USA), CD9 (sc-13118, SantaCruz, Dallas, TX, USA), Calnexin (sc-11397, SantaCruz), and GAPDH (sc-47724, Santa Cruz) were applied in a 1:1000 dilution. Secondary horseradish peroxidase-conjugated antibodies (1:40.000; anti-rabbit: sc2004 and anti-mouse: sc2005) and the chemiluminescence Amersham ECL reaction kit (GE Healthcare, Uppsala, Sweden) were used for detection.

### 4.17. Pathway Analysis

Pathway analysis of radiation-deregulated miRNAs and proteins was performed by the software tool Ingenuity Pathway Analysis (IPA Ingenuity System, http://www.Ingenuity.com).

Availability of data and materials: Sequencing data were deposited under STUDY PRJEB35150 at https://www.ebi.ac.uk/ena. The raw MS data were deposited in the RBstore database, Study ID: 1155: (https://www.storedb.org/store_v3/create_dataset.jsp?studyId=1155).

## Supplementary Materials

Supplementary materials can be found at https://www.mdpi.com/1422-0067/21/7/2336/s1.
