# Supplementary figures and images for: Radiation Exposure of Peripheral Mononuclear Blood Cells Alters the Composition and Function of Secreted Extracellular Vesicles
Source: Int J Mol Sci. 2020 Mar 27;21(7):2336. doi: 10.3390/ijms21072336 (PMC7178185; doi:10.3390/ijms21072336)

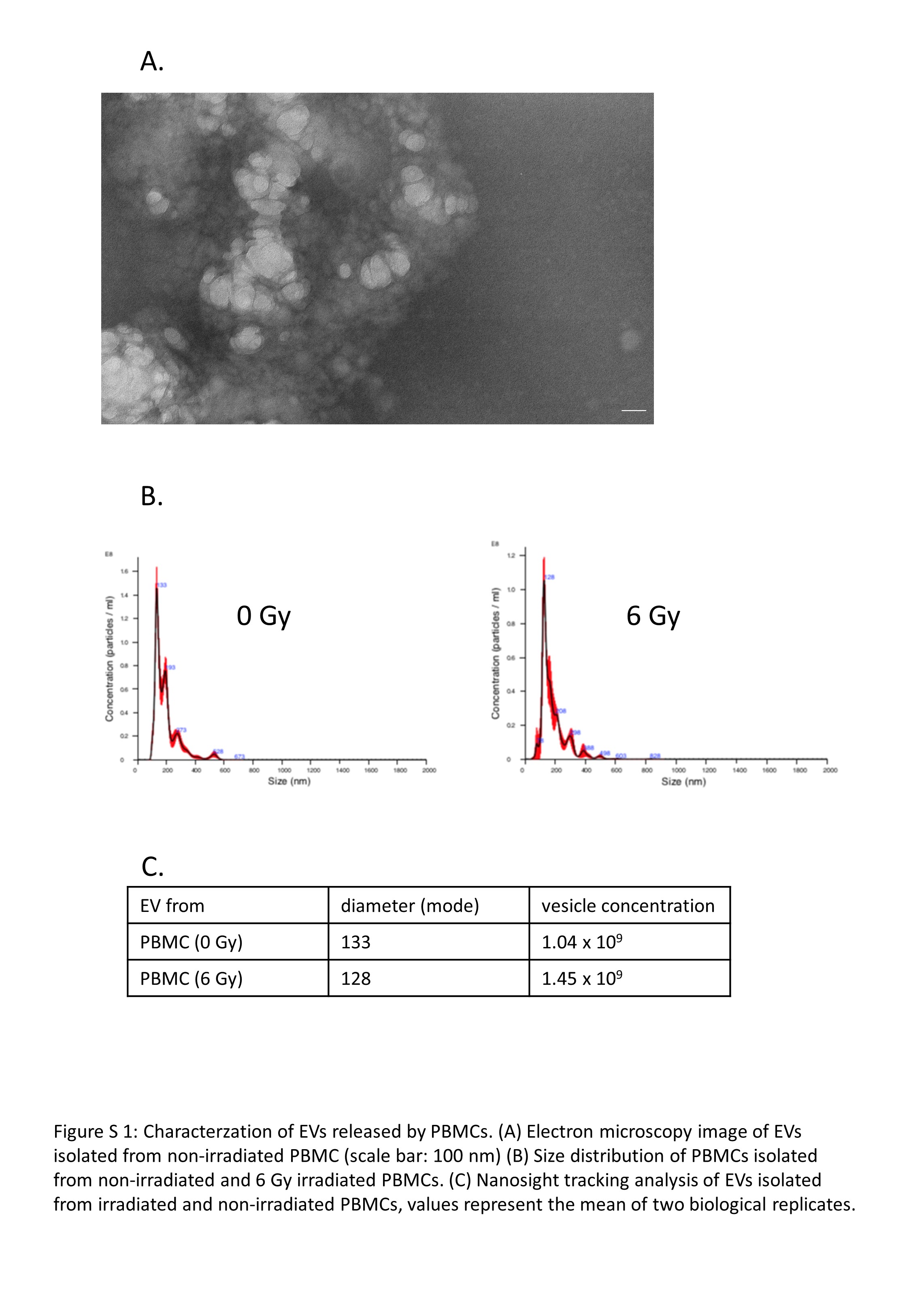

Supplement: Supplementary file 1 [file ijms-21-02336-s001.zip › Supplementary_S1.jpg]

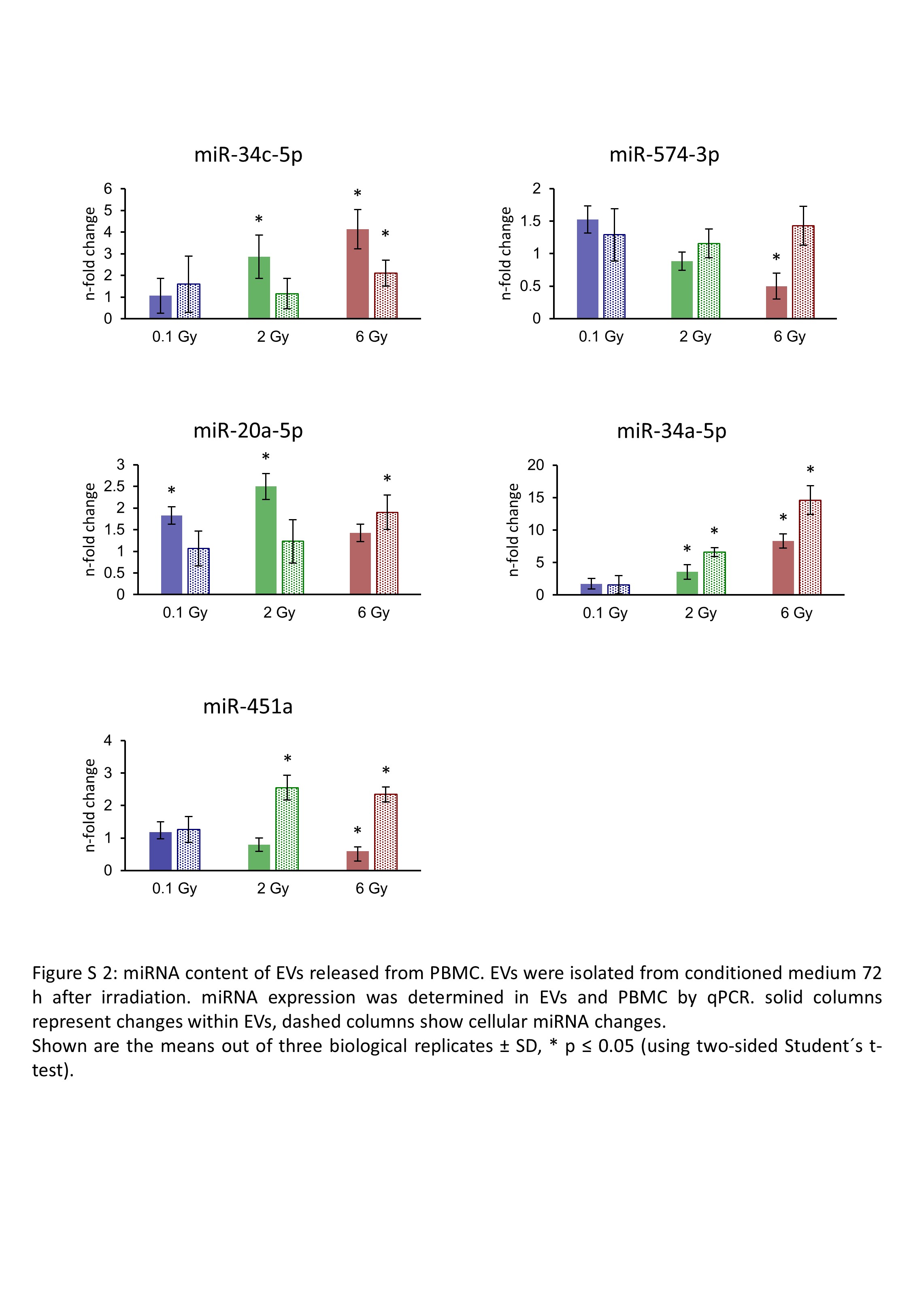

Supplement: Supplementary file 1 [file ijms-21-02336-s001.zip › Supplementary_S2.jpg]

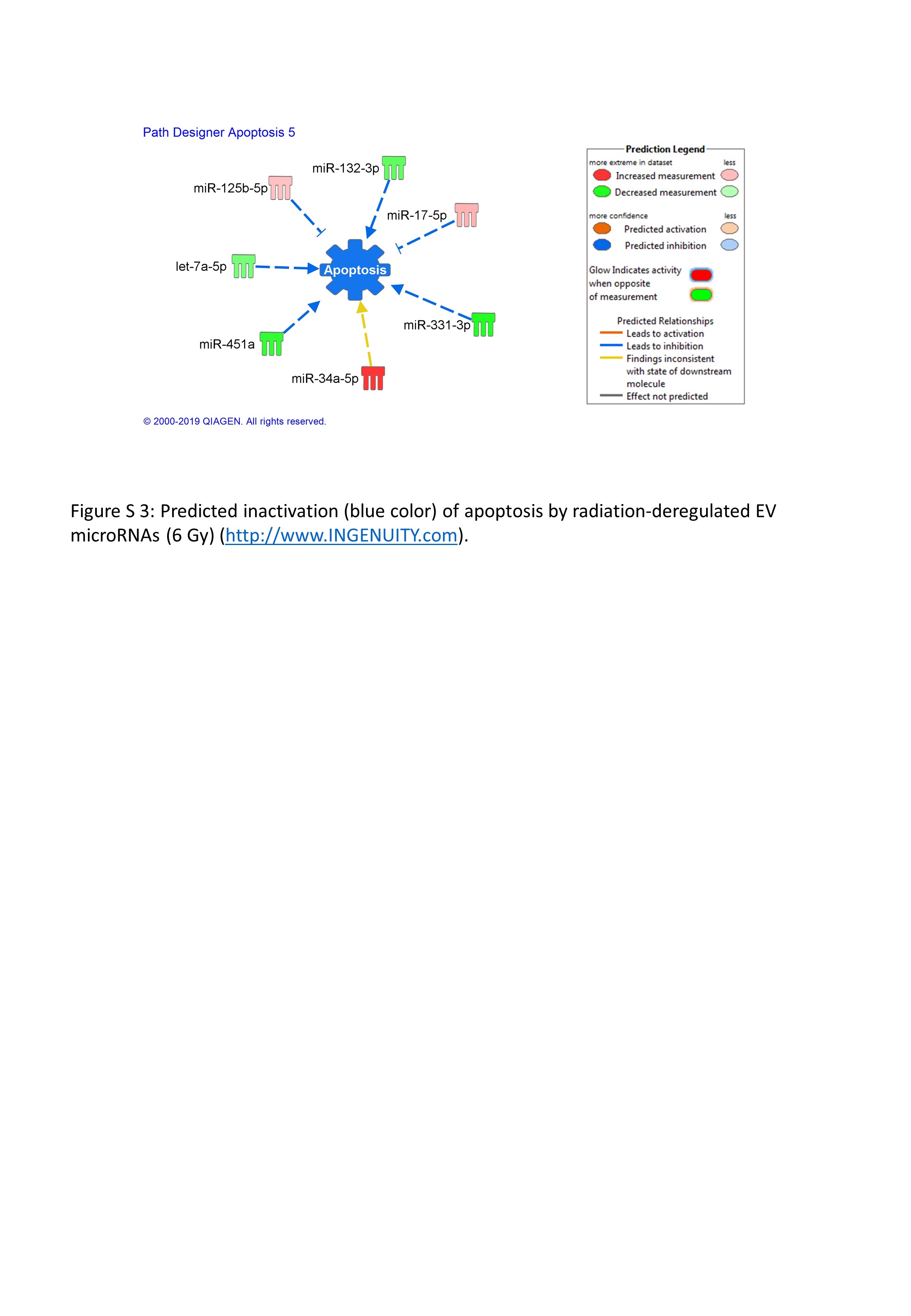

Supplement: Supplementary file 1 [file ijms-21-02336-s001.zip › Supplementary_S3.jpg]

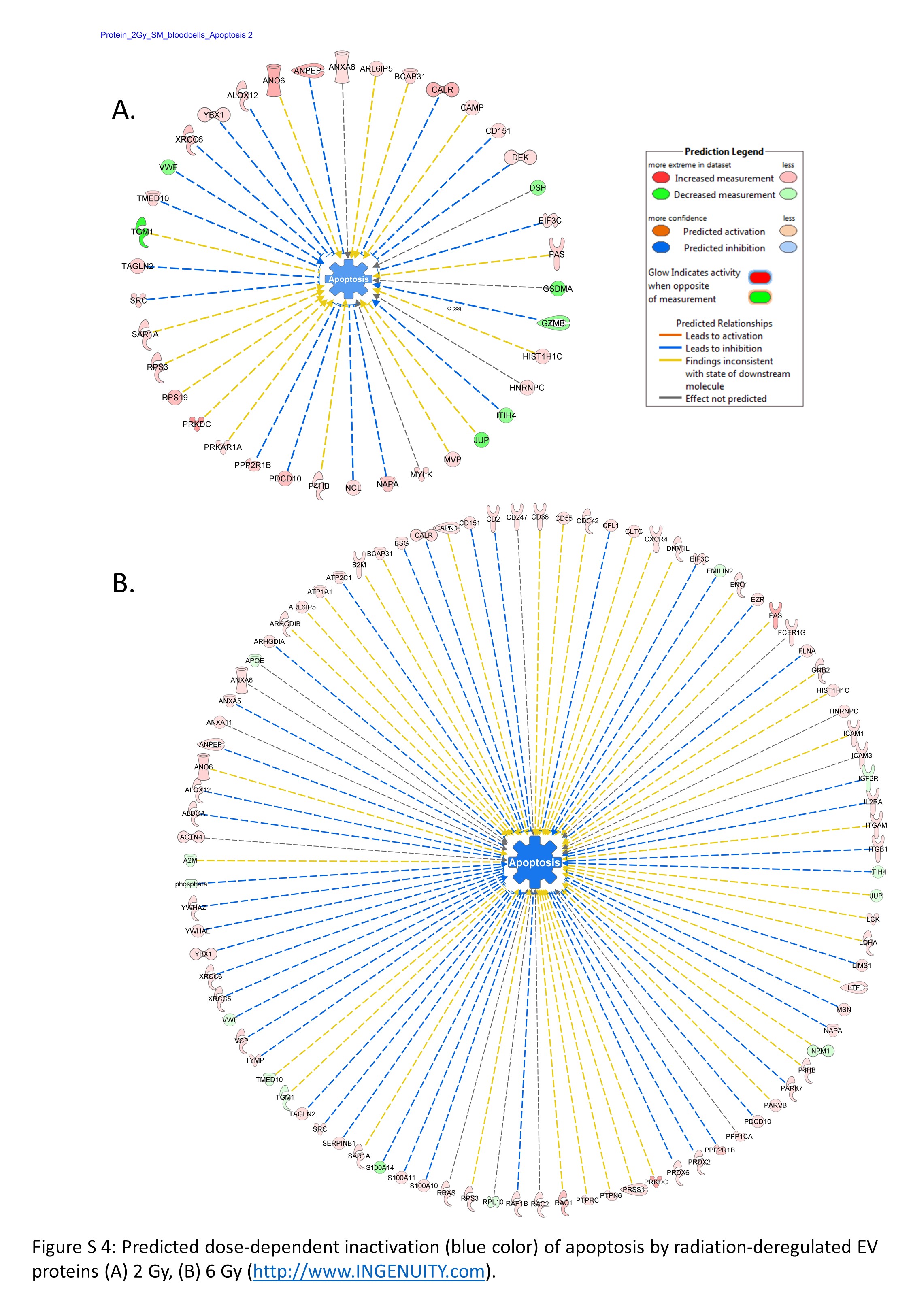

Supplement: Supplementary file 1 [file ijms-21-02336-s001.zip › Supplementary_S4.jpg]

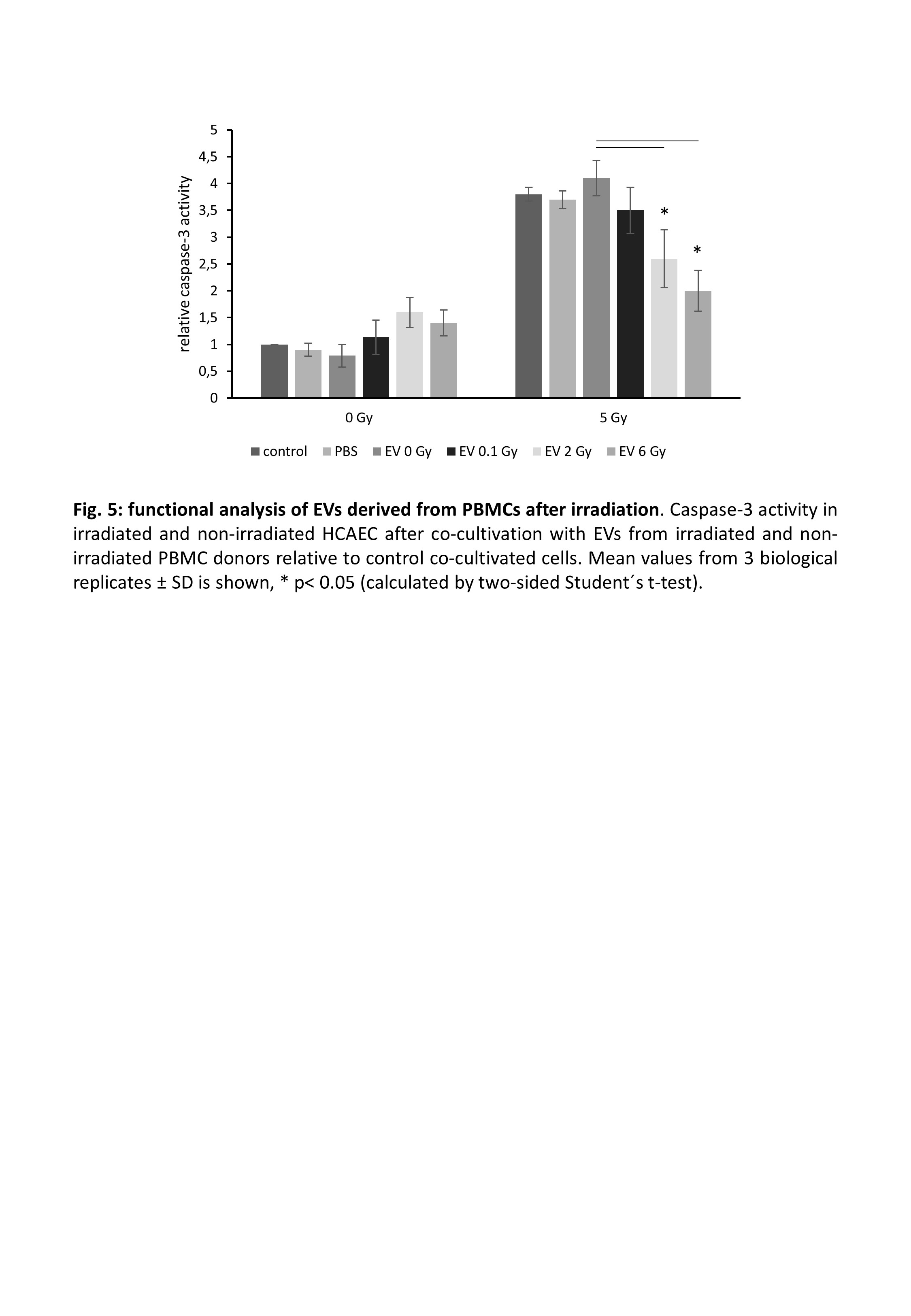

Supplement: Supplementary file 1 [file ijms-21-02336-s001.zip › Supplementary_S5.jpg]
